# A prospective multicenter study on varicella-zoster virus infection in children with acute lymphoblastic leukemia

**DOI:** 10.3389/fcimb.2022.981220

**Published:** 2022-11-11

**Authors:** Peifang Xiao, Jiaoyang Cai, Ju Gao, Wei Gao, Xianmin Guan, Alex Wing Kwan Leung, Yiying He, Yong Zhuang, Jinhua Chu, Xiaowen Zhai, Benquan Qi, Aiguo Liu, Liangchun Yang, Jiashi Zhu, Zheng Li, Xin Tian, Yao Xue, Li Hao, Xuedong Wu, Fen Zhou, Lingzhen Wang, Jingyan Tang, Shuhong Shen, Shaoyan Hu

**Affiliations:** ^1^ Department of Hematology, Jiangsu Children Hematology and Oncology Center Children’s Hospital of Soochow University, Suzhou, China; ^2^ Department of Hematology/Oncology, Shanghai Children’s Medical Center, School of Medicine, Shanghai Jiao Tong University, National Health Committee Key Laboratory of Pediatric Hematology and Oncology, Shanghai, China; ^3^ Department of Pediatrics, West China Second University Hospital, Sichuan University, Key Laboratory of Birth Defects and Related Disease of Women and Children, Ministry of Education, Chengdu, China; ^4^ Department of Hematology/Oncology, Chongqing Medical University Affiliated Children’s Hospital, Chongqing, China; ^5^ Department of Pediatrics, Hong Kong Children’s Hospital, The Chinese University of Hong Kong, Hong Kong, Hong Kong SAR, China; ^6^ Department of Hematology/Oncology, Guangzhou Women and Children’s Medical Center, Guangzhou, China; ^7^ Department of Pediatrics, Qilu Hospital of Shandong University, Jinan, China; ^8^ Department of Pediatrics, Anhui Medical University Second Affiliated Hospital, Anhui, China; ^9^ Department of Hematology/Oncology, Children’s Hospital of Fudan University, Shanghai, China; ^10^ Department of Pediatrics, State Key Laboratory of Experimental Hematology, National Clinical Research Center for Blood Diseases, Institute of Hematology and Blood Diseases Hospital, Chinese Academy of Medical Sciences and Peking Union Medical College, Tianjin, China; ^11^ Department of Pediatrics, Tongji Hospital of Tongji Medical College, Huazhong University of Science and Technology, Wuhan, China; ^12^ Department of Pediatrics, Xiangya Hospital Central South University, Changsha, China; ^13^ Department of Hematology/Oncology, Children’s Hospital Affiliated to Shanghai Jiao Tong University, Shanghai, China; ^14^ Department of Hematology/Oncology, Jiangxi Provincial Children’s Hospital, Nanchang, China; ^15^ Department of Hematology/Oncology, KunMing Children’s Hospital, Kunming, China; ^16^ Department of Hematology/Oncology, Children’s Hospital of Nanjing Medical University, Nanjing, China; ^17^ Department of Hematology/Oncology, Xi’an Northwest Women’s and Children’s Hospital, Xi’an, China; ^18^ Department of Pediatrics, Nanfang Hospital, Southern Medical University, Guangzhou, China; ^19^ Department of Pediatrics, Xiehe Hospital of Tongji Medical College, Huazhong University of Science and Technology, Wuhan, China; ^20^ Department of Pediatrics, The Affiliated Hospital of Qingdao Medical University, Qingdao, China

**Keywords:** childhood, acute lymphoblastic leukemia, varicella-zoster infection, *E2A/PBX1* fusion gene, risk factor

## Abstract

**Background and methods:**

The study evaluated prognostic factors associated with varicella-zoster virus (VZV) infection and mortality in children with acute lymphoblastic leukemia (ALL) using data from the multicenter Chinese Children’s Cancer Group ALL-2015 trial.

**Results:**

In total, 7,640 patients were recruited, and 138 cases of VZV infection were identified. The incidence of VZV infection was higher in patients aged ≥ 10 years (22.5%) and in patients with the *E2A/PBX1* fusion gene (11.6%) compared to those aged < 10 years (13.25%, *P* = 0.003) or with other fusion genes (4.9%, *P* = 0.001). Of the 10 deaths in children with ALL and VZV infection, 4 resulted from VZV complications. The differences between groups in the 5-year overall survival, event-free survival, cumulative recurrence, and death in remission were not statistically significant. The proportion of complex infection was higher in children with a history of exposure to someone with VZV infection (17.9% vs. 3.6%, *P* = 0.022).

**Conclusion:**

VZV exposure was associated with an increased incidence of complex VZV infection and contributed to VZV-associated death in children with ALL.

## Introduction

Acute lymphoblastic leukemia (ALL) is the most common childhood malignancy. The 5-year survival rate for childhood ALL in developed countries exceeds 90% (Hunger et al., 2012; Pui et al., 2017; Kowalczyk et al., 2019). In patients with ALL, age, sex, race, National Cancer Institute stratification, and immunophenotype are independent predictors of outcome (Hunger et al., 2012). Data from the Chinese Children’s Cancer Group ALL-2015 (CCCG-ALL-2015) trial (registration number: ChiCTR-IPR-14005706) showed that the 5-year survival rate for ALL exceeded 91%, with a 95.2% 5-year survival rates in patients in the standard-risk group (Tang et al., 2021). Relapse events and treatment-related complications, including infection and toxicity, are the main factors affecting survival (Oskarsson et al., 2018; Hunger and Raetz, 2020). In recent years, the maturation and widespread application of chimeric antigen receptor T-cell technologies, and the application of monoclonal antibodies and hematopoietic stem cell transplantation in the treatment of high-risk and recurrent childhood ALL, has resulted in a significant reduction in mortality rates and improved survival (Maude et al., 2018; Tran and Hunger, 2020; Jasinski et al., 2020; Zhao et al., 2021). The prevention of treatment-related complications and resultant mortality has become a core treatment consideration. Previous studies have shown that 30−47% of deaths in children with ALL were treatment-related (O'Connor et al., 2014; Pui et al., 2014; Cui et al., 2018). Infection is the most common complication of chemotherapy and a predominant cause of treatment-related death (Hough and Vora, 2017). The prevention and treatment of infection is therefore key to improving the long-term survival of children with ALL.

Varicella-zoster virus (VZV) causes both varicella (chickenpox) and herpes zoster (HZ), also known as shingles. Varicella is a disease and does not reactivate, whilst VZV reactivates and causes HZ. In healthy children, varicella is a benign, self-limiting disease, whereas in immunocompromised children, VZV infection can be life-threatening. ALL suppresses the immune system, and chemotherapeutic agents such as steroids, methotrexate, and 6-mercaptopurine, can further reduce the B lymphocyte, T lymphocyte, and total immunoglobulin levels in children with ALL (Hill et al., 2005), making them vulnerable to VZV infection. The results of 2 previous multicenter studies (Hill et al., 2005; Zawitkowska et al., 2020) indicated that the incidence of VZV infection in patients with ALL has decreased significantly with the increased accessibility to vaccines. The adverse impact of VZV on the prognosis of ALL has also decreased. However, to date, no prospective studies have examined the clinical and prognostic importance of VZV infection in a large cohort of Asian children with ALL.

The CCCG-ALL-2015 trial was conducted in 20 major hospitals and medical centers in China, located in 18 provinces and municipalities, covering three-quarters of the country. This trial included Han Chinese and ethnic minorities such as Miao, Bai, Yi, and Tibetans. The rate of VZV vaccination and treatments administered to patients with varicella varied by region. Therefore, the study of VZV infection in children treated according to the CCCG-ALL-2015 protocol and impact of VZV infection on the prognosis of children with ALL is instructive in assessing its effectiveness, safety, and generalizability.

## Methods

### Ethics approval and study design

This study was conducted in accordance with the Declaration of Helsinki and national and institutional ethics standards. This study was approved by the institutional review board of each participating center following the Central Institutional Review Board (approval number: SCMCIRB-K2014060). Informed consent was obtained from parents, guardians, or patients, as appropriate.

### Case data

The information of each patient was required to be record in the case report form (CRF). Information on all cases of VZV infection was recorded as a part of adverse events and severe adverse events reporting, and detailed information related to VZV infection was collected, as specified in the protocol (ChiCTRIPR-14005706). Data on VZV infection was collected using a CRF.

### Diagnosis and classification

Diagnosis and classification of ALL were based on the morphology, cytogenetics, immunology, and molecular biology of leukemia cells. The fusion genes were identified by multiplex RT-PCR. The risk criteria were noted in the trial and all patients were treated according to the risk classification (Tang et al., 2021). VZV infection was diagnosed mainly based on the presence of typical skin manifestations which was further confirmed using polymerase chain reaction (PCR) tests which detected VZV in vesicular fluid samples. VZV infection was classified as simple or complicated based on whether there was organ involvement. Primary infection with VZV causes varicella, and whereas the rash of herpes zoster is distributed along the nerves, varicella causes a rash which is typically distributed throughout the body. Further details are outlined in the CCCG-ALL-2015 protocol.

### Study design and outcomes

Investigating VZV infection and its prognostic relevance in children with ALL was one of the main purposes of the CCCG-ALL 2015 trial. In the trial protocol, VZV infection was well-defined, and treatment procedures were standardized. This study aimed to investigate the incidence and clinical characteristics of children with ALL and VZV infection, and its impact on disease prognosis and outcome.

The primary endpoint was the occurrence of varicella/HZ. The secondary endpoints were event-free survival (EFS), overall survival (OS), and recurrence-free survival (RFS). OS refers to the time from disease diagnosis to death or the last follow-up. EFS refers to the time from diagnosis to induction failure, relapse, death, drop out, off-protocol treatment at the discretion of the treating physician, or the development of a second malignancy. The cumulative recurrence rate (CRR) refers to the cumulative incidence of local recurrence or metastases in all patients since initial remission. RFS refers to the survival time from initial remission to local recurrence or tumor metastasis. Outcome data reported here were updated on June 30, 2021.

### Treatment

Patients received multi-agent chemotherapy at the following treatment phases: induction (weeks 1–7), consolidation (weeks 8–15), continuation therapy and reinduction (weeks 16–34), and maintenance phase (weeks 35–125) (Tang et al., 2021). Treatment for VZV mainly included isolation until skin scab formation, followed by intravenous immunoglobulin (IVIG) and/or acyclovir.

### Statistical analysis

Survival and competing risk statistical analyses were conducted using the R statistical software package, version 3.4.4 (R Foundation for Statistical Computing, Vienna, Austria; https://www.r-project.org/). Other data were analyzed using SPSS Statistics for Windows, version 20.0 (IBM Corp., Armonk, NY, USA). Count data were presented as frequencies with percentages (%). Pearson’s Chi-squared (χ^2^) test was used to compare differences between groups when the total number of patients was > 40 and the expected frequency was T ≥ 5, whereas Yates continuity correction of χ^2^ was used when the total was > 40 and 1 ≤ T < 5. Fisher’s exact test was used when the total was < 40, the minimal expected frequency was < 1, or the *P* value was close to the inspection level (α = 0.05). In the case-control study, odds ratios (ORs) and 95% confidence intervals (CIs) were calculated using logistic regression. Variables with *P* < 0.20 at the univariate level were entered into a multivariable logistic regression model.

EFS, RFS, and OS curves were estimated using the Kaplan-Meier method and groups were compared using the log-rank test. Multivariable analyses were performed using Cox regression to assess the independent predictors of EFS. Standard errors were estimated using Peto’s method. The cumulative incidence of relapse events or toxicity-related deaths was estimated using the method of Kalbfleisch and Prentice and compared using Gray’s test to account for competing events. Competing events for relapse included death during remission, second malignancy, off-protocol treatment by decision of the treating physician, treatment abandonment, and transfer to another hospital. Competing events for death during remission included any relapse, second malignancy, and off-protocol treatment by the decision of the treating physician. CIs were calculated with a large-sample normal approximation. Two-tailed *P*-values < 0.05 were considered statistically significant.

## Results

### Clinical characteristics of children with acute lymphoblastic leukemia and varicella-zoster virus infection

From January 1, 2015 to December 31, 2019, 7,640 patients were newly diagnosed with pediatric ALL, and 138 patients developed VZV infection. No patients were repeatedly infected. The incidence of VZV infection was 1.8%. Of the 138 patients with VZV infection, 128 survived and 10 died. Among the 10 patients who died, 4 resulted from complex VZV (4/10,40%), accounting for 2.9% (4/138) of the total cases of VZV infection, and 0.05% (4/7640) of newly ALL cases. The characteristics of the children in the ALL cohort, with and without VZV infection, are described in **Table 1**. The patients with VZV infection were predominantly male (n = 81; 58.7%), with a median age of 6.4 years (range: 1.1–16.2 years). Patients aged ≥ 10 years had a nearly two-fold higher incidence of VZV (3.0%) than younger patients (1.6%) (*P* = 0.003). Additionally, *E2A/PBX1* positivity was more prevalent in the patients with VZV infection (4.2%) than in those without VZV infection (1.7%; *P* = 0.001). Sex, immune type, other subtypes classified based on other fusion genes, and risk classification did not differ significantly between the 2 groups (**Table 1**). Multivariable logistic regression analysis identified age ≥ 10 years and *E2A/PBX1* positivity as independent risk factors for VZV infection in children with ALL (**Supplementary Table S1**).

**Table 1:** Characteristics of children with acute lymphoblastic leukemia, with and without and varicella-zoster virus infection.

Group	Without VZV infection (N = 7502)(n, %)	With VZV infection (N = 138)(n, %)	OR (95% CI)	*P* value
Age (years)				0.003
< 10	6508 (98.4)	107 (1.6)	1.00 (ref.)	
≥ 10	994 (97.0)	31 (3.0)	1.90 (1.27–2.85)	
Sex				0.930
Male	4440 (98.2)	81 (1.8)	1.00 (ref.)	
Female	3062 (98.2)	57 (1.8)	1.02 (0.73–1.44)	
Immunotype				0.660
B-cell ALL	6793 (98.2)	123 (1.8)	1.00 (ref.)	
T-cell ALL	709 (97.9)	15 (2.1)	1.17 (0.68–2.01)	
*TEL/AML1*				0.300
Positive	1327 (98.7)	18 (1.3)	1.00 (ref.)	
Negative	6175 (98.1)	120 (1.9)	1.32 (0.80–2.17)	
*E2A/PBX1*				0.001
Positive	363 (95.8)	16 (4.2)	1.00 (ref.)	
Negative	7139 (98.3)	122 (1.7)	0.39 (0.23–0.66)	
*BCR/ABL1*				0.944
Positive	317 (98.1)	6 (1.9)	1.00 (ref.)	
Negative	7185 (98.2)	132 (1.8)	0.97 (0.43–2.22)	
*MLL-r*				0.930
Positive	155 (98.1)	3 (1.9)	1.00 (ref.)	
Negative	7347 (98.2)	135 (1.8)	0.95 (0.30–3.01)	
Risk				0.169
Low	3885 (98.4)	63 (1.6)	1.00 (ref.)	
Intermediate/high	3617 (98.0)	75 (2.0)	1.28 (0.91–1.79)	
Treatment phase				<0.001
Induction	7502	22		
Consolidation	7246	14		
Continuation therapy 1	7075	38		
Continuation therapy 2	6766	63		
Completion of chemotherapy	5198	1		

CI, confidence interval; OR, odds ratio; VZV, varicella-zoster virus.

Of the 7640 ALL patients, 3818 patients (49.9%) received one dose of the vaccine before being diagnosed with ALL, and 92 of the 138 patients with VZV infection were vaccinated. Twenty-two patients (15.9%) were in induced remission, 14 (10.1%) were in the consolidation stage, 38 (27.5%) were undergoing continuation therapy 1, 63 (45.7%) were undergoing continuation therapy 2, and 1 (0.7%) was at the end of treatment (**Table 2**). Chemotherapy was delayed in 121 patients because of VZV infection. Nine patients had confirmed contact with someone with varicella; and 109 patients had accurate treatment records (**Table 2**).

**Table 2:** Comparison of the clinical features of skin only and complicated varicella-zoster virus infection in children with acute lymphoblastic leukemia.

Factor	Skin only (N = 110)n (%)	Complicated (N = 28)(n, %)	OR (95% CI)	*P* value
Sex				0.270
Female	62 (56.4)	19 (68)	1.00 (ref.)	
Male	48 (43.6)	9 (32)	0.61 (0.25–1.47)	
Age (years)				0.245
<10	83 (75.5)	24 (86)	1.00 (ref.)	
≥10	27 (24.5)	4 (14)	0.51 (0.16–1.61)	
Immunotype				0.322
B-cell ALL	100 (90.9)	23 (82)	1.00 (ref.)	
T-cell ALL	10 (9.1)	5 (18)	2.17 (0.68–6.97)	
Therapy^*^				0.061
Acyclovir	25 (30.9)	5 (18)		
IVIG	19 (23.5)	3 (11)		
Acyclovir and IVIG	37 (45.7)	20 (71)		
Varicella vaccination				0.052
Yes	69 (62.7)	23 (82)	1.00 (ref.)	
No	41 (37.3)	5 (18)	0.37 (0.13–1.04)	
Treatment phase				0.891
Induction	18 (16.4)	4 (14)		
Consolidation	12 (10.9)	2 (7)		
Continuation therapy 1	29 (26.4)	9 (32)		
Continuation therapy 2	50 (45.5)	13 (46)		
Completion of chemotherapy	1 (0.9)	0 (0)		
Fusion gene^†^				0.560
Positive	33 (30.0)	10 (36)	1.00 (ref.)	
Negative	77 (70.0)	18 (64)	0.771 (0.322–1.849)	
Risk				0.453
Low	51 (46.4)	12 (43)		
Intermediate	56 (50.9)	16 (57)		
High	3 (2.7)	0 (0)		
Treatment delay				0.541
Yes	95 (86.4)	26 (93)	1.00 (ref.)	
No	15 (13.6)	2 (7)	0.77 (0.32–1.85)	
Karyotype^‡^				0.367
Hypo	4 (4.0)	1 (4)		
Hyper	26 (26.3)	3 (13)		
Normal	69 (69.7)	19 (83)		
Exposure				0.022
Yes	4 (3.6)	5 (18)	1.00 (ref.)	
No	106 (96.4)	23 (82)	0.17 (0.04–0.70)	
5 years OS	(93.4% ± 2.9%)	(59.4% ± 24.6%)		0.040
5 years RFS	(89.9% ± 3.3%)	(79.2% ± 13.0%)		0.548

^†^Fusion gene including TEL/AML1, E2A/PBX1, BCR/ABL, MLL-related (MLL-r).

^‡^Among patients with data available.

ALL, acute lymphoblastic leukemia; CI, confidence interval; IVIG, intravenous immunoglobulin; OR, odds ratio; OS, overall survival; RFS, recurrence-free survival; VZV, varicella-zoster virus.

VZV infections were classified as simple type (skin lesions only, without bacterial infection) in 110 patients (79.7%) and complex type (skin lesions with various infections or other organs affected) in 28 patients (20.3%). The subgroup with complex type VZV infection included 17 cases (60.7%) of cutaneous varicella with skin infection, 6 cases (21.4%) of cutaneous varicella combined with pneumonia, one case (1.9%) of cutaneous varicella combined with encephalitis, one (1.9%) of cutaneous varicella combined with respiratory and urinary tract involvement, one case (1.9%) of cutaneous varicella combined with skin infection and central nervous system involvement, one case (1.9%) of cutaneous varicella combined with septicemia, and one case (1.9%) with complications but no specific organ affected (**Table 2**).

Children with VZV exposure history were significantly more likely to develop complex VZV infections. The mortality rate was higher among children with complex VZV infection (14.3%) with all death cases being VZV-related, whereas that of simple VZV infection was only 5.45%, including 5 cases of ALL relapses and one of hemophagocytic lymphohistiocytosis. None of the patients died of simple VZV infection.

Among the 138 children with ALL and VZV infection there were 106 cases of varicella and 32 cases of HZ (**Table 3**). Patients aged < 10 years were more likely to present with varicella after VZV infection (83.2% vs. 54.8%), whereas children aged ≥ 10 years were more likely to develop HZ (45.2% vs. 16.8%; *P* = 0.001). However, the 5-year OS and RFS rates were similar between children with varicella and those with HZ (**Table 3**).

**Table 3:** Characteristics of children with acute lymphoblastic leukemia and varicella or herpes zoster.

Factor	Varicella (N = 106)(n, %)	Herpes zoster (N = 32)(n, %)	OR (95% CI)	*P* value
Sex				0.117
Female	43 (40.6)	18 (56)	1.00 (ref.)	
Male	63 (59.4)	14 (44)	0.53 (0.24–1.18)	
Age (years)				0.001
< 10	89 (84.0)	18 (56)	1.00 (ref.)	
≥ 10	17 (16.0)	14 (44)	4.07 (1.71–9.72)	
Immunotype				>0.999
B-cell ALL	94 (88.7)	29 (91)	1.00 (ref.)	
T-cell ALL	12 (11.3)	3 (9)	0.81 (0.21–3.07)	
Therapy^†^				0.008
Acyclovir	17 (20.2)	13 (52)		
IVIG	19 (22.6)	3 (12)		
Acyclovir and IVIG	48 (57.1)	9 (36)		
Varicella vaccination				0.568
Yes	72 (67.9)	20 (62.5)	1.00 (ref.)	
No	34 (32.1)	12 (37.5)	1.06 (0.45–2.51)	
Treatment phase				0.053
Induction	17 (16.0)	5 (16)		
Consolidation	14 (13.2)	0 (0)		
Continuation therapy 1	26 (24.5)	12 (37.5)		
Continuation therapy 2	48 (45,3)	15 (47)		
Completion of chemotherapy	1 (0.9)	0 (0)		
Treatment delay				0.116
Yes	96 (90.6)	25 (78)	1.00 (ref.)	
No	10 (9.4)	7 (22)	2.70 (0.93–7.77)	
Fusion gene^‡^				0.391
Positive	35 (33.0)	8 (25)	1.00 (ref.)	
Negative	71 (67.0)	24 (75)	1.48 (0.60–3.63)	
Risk				0.247
Low	50 (47.2)	13 (41)		
Intermediate	55 (51.9)	17 (53)		
High	1 (0.9)	2 (6)		
Karyotype				0.761
Hypo	3 (2.8)	2 (6)		
Hyper	21 (19.8)	8 (25)		
Normal	66 (62.3)	22 (69)		
Exposure				0.632
Yes	8 (7.5)	1 (3)	1.00 (ref.)	
No	98 (92.5)	31(97)	2.53 (0.30–21.04)	
5-year OS (%)	89.6 ± 3.2	92.8 ± 4.9		0.578
5-year RFS (%)	86.9 ± 3.6	86.9 ± 6.1		0.880

^†^Among patients with data available.

^‡^Fusion gene including TEL/AML1, E2A/PBX1, BCR/ABL, MLL-related (MLL-r).

CI, confidence interval; IVIG, intravenous immunoglobulin; OR, odds ratio; OS, overall survival; RFS, recurrence-free survival.

### Outcomes of patients with acute lymphoblastic leukemia and varicella-zoster virus infection

In total, 10 (7.2%) of the 138 patients with VZV infection died during the follow-up period, of whom 7 (70%) were male; 9 had B-cell ALL, and 1 had T-cell ALL. Four patients died of complications of VZV infection, 5 patients died of relapse, and 1 died of treatment-related toxicity. Two relapses occurred after completion of chemotherapy and 3 relapsed during continuation stage 1 or 2. One patient infected with VZV who died was in the consolidation stage of ALL treatment, 2 were infected during induction therapy, and 7 were infected during the continuation stage. Four patients were classified as low-risk, 5 as intermediate-risk, and 1 as high-risk. Among the low-risk patients, only one patient died of an ALL relapse. Four patients died of severe infections secondary to complicated VZV infection with a confirmed history of VZV exposure, one died of hemophagocytic lymphohistiocytosis (as simple VZV infection had been under control for 15 months), and the other 5 patients with simple VZV died of ALL relapse (**Table 4**).

**Table 4:** Detailed information of patients died either VZV infection or other causes.

Case	Cause of death	VZV DNA	Outcome of VZV Infection	Stage of therapy at time of death	Time to VZV onset (d)	D19MRD	D46MRD	Age (months)	Sex	Fusion	Treatment delay	Clinical type	Stage of therapy at time of onset	Complicated	Immunotype	Vaccination	Risk	Exposure
1	VZV Infection	Positive	Death	Continuation therapy 1	0	0.0116	0	69	Female	*TEL/AML1*	Yes	Varicella	Continuation therapy 1	Yes	B-cell ALL	Yes	Low	Yes
2	Relapse	Positive	Improvement	Continuation therapy 2	19	0.59	0	140	Male	Negative	Yes	Varicella	Induction	No	T-cell ALL	Yes	Intermediate	No
3	VZV Infection	Positive	Death	Continuation therapy 2	0	0	0.09	94	Female	Negative	Yes	Varicella	Continuation therapy 2	Yes	B-cell ALL	Yes	Intermediate	Yes
4	Relapse	Positive	Improvement	Continuation therapy 1	11	0	0	70	Male	*E2A/PBX1*	Yes	Varicella	Induction	Yes	B-cell ALL	Yes	Intermediate	No
5	Relapse	Positive	Improvement	Completing chemotherapy	39	0.62	0.29	97	Male	*BCR/ABL1*	Yes	Varicella	Continuation therapy 2	Yes	B-cell ALL	Yes	Intermediate	No
6	VZV Infection	Positive	Death	Consolidation	0	0.03	0	136	Male	*E2A/PBX1*	Yes	Varicella	Consolidation	Yes	B-cell ALL	Yes	Intermediate	Yes
7	VZV Infection	Positive	Death	Continuation therapy 1	0	52.4	0.01	33	Male	Negative	Yes	Herpes zoster	Continuation therapy 1	Yes	B-cell ALL	No	Intermediate	Yes
8	Relapse	Positive	Improvement	Continuation therapy 2	20	0.88	0	180	Male	Negative	Yes	Herpes zoster	Continuation therapy 1	No	B-cell ALL	Yes	Low	No
9	Relapse	Positive	Improvement	Completing chemotherapy	16	45.37	—	130	Male	Negative	Yes	Herpes zoster	Continuation therapy 2	No	B-cell ALL	No	High	No
10	Treatment-related	Positive	Improvement	Continuation therapy 2	15	0	0	13	Female	Negative	Yes	Herpes zoster	Continuation therapy 2	No	B-cell ALL	Yes	Low	No

19MRD, minimal residual disease on 19th day of induction chemotherapy; D46MRD, minimal residual disease on 46th day of induction chemotherapy; VZV, varicella-zoster virus.

Among the 138 children with ALL and VZV infection, 17 experienced relapses, the majority of whom were male (**Supplementary Table S2**). None of the factors potentially influencing OS and RFS were statistically significant (**Table 5**). However, immunophenotype, initial white blood cell count, risk classification, and *BCR/ABL1* gene fusion were prognostic for EFS (all *P* < 0.05). Patients with T-ALL, WBC ≥ 50×10^9^/L, intermediate/high risk, and *BCR/ABL1* gene fusion had lower EFS (**Table 5**). Immunophenotype was an independent prognostic factor for EFS based on the multivariable analysis (*P* = 0.029) (**Supplementary Table S3**).

**Table 5:** Factors influencing the 5-year overall survival, recurrence-free survival, and event-free survival in patients with varicella-zoster virus infection.

Factor	No. of patients	5-year OS (%)	*P*	5-year RFS (%)	*P*	5-year EFS (%)	*P*
Age (years)			0.283		0.291		0.22
1–10	107	91.6 ± 2.9		88.1 ± 3.5		82.0 ± 4.1	
>10	31	85.1 ± 7.0		82.6 ± 7.1		72.9 ± 8.3	
Sex			0.988		0.409		0.519
Male	81	90.0 ± 3.7		852 ± 4.2		78.8 ± 4.8	
Female	57	91.1 ± 3.8		89.0 ± 5.0		81.8 ± 5.8	
Immunotype			0.079		0.211		0.026
B-ALL	123	91.8 ± 2.7		87.9 ± 3.2		83.1 ± 3.6	
T-ALL	15	75.7 ± 12.4		76.2 ± 12.1		56.6 ± 13.8	
WBC (×10^9^/L)			0.06		0.066		0.013
<50	109	90.2 ± 2.4		90.2 ± 3.0		84.7 ± 3.5	
≥50	29	77.6 ± 9.2		73.0 ± 10.1		60.8 ± 11.1	
Initial risk			0.2		0.084		0.027
Low	70	94.1 ± 2.9		92.4 ± 3.3		88.1 ± 4.0	
Intermediate/high	68	86.1 ± 4.7		81.0 ± 5.4		71.6 ± 6.1	
Ultimate risk			0.374		0.074		0.042
Low	63	93.4 ± 3.2		93.2 ± 3.3		88.4 ± 4.1	
Intermediate/high	75	87.6 ± 4.2		81.5 ± 5.0		73.1 ± 5.6	
Karyotype			0.960		0.892		0.816
>50	23	90.2 ± 3.0		87.2 ± 3.4		79.8 ± 4.1	
Others	115	90.9 ± 6.1		85.9 ± 7.6		81.6 ± 8.3	
*TEL/AML1*			0.635		0.388		0.366
Positive	18	94.4 ± 5.4		92.9 ± 6.9		87.7 ± 8.2	
Negative	120	86.9 ± 3.1		85.9 ± 3.5		78.7 ± 4.1	
*E2A/PBX1*			0.469		0.75		0.793
Positive	16	87.5 ± 8.3		87.5 ± 8.3		81.3 ± 9.8	
Negative	122	90.8 ± 2.8		87.0 ± 3.3		80.0 ± 3.9	
*BCR/ABL1*			0.551		0.1		0.004
Positive	6	75.0 ± 21.7		37.5 ± 28.6		31.3 ± 24.5	
Negative	132	91.2 ± 2.5		88.7 ± 2.9		82.1 ± 3.5	
*MLL-r*			0.584		0.517		0.415
Positive	3	100		100		100	
Negative	135	90.1 ± 2.8		86.5 ± 3.2		79.5 ± 3.8	
Treatment delay			0.208		0.507		0.506
Yes	121	89.1 ± 3.1		86.2 ± 3.4		79.2 ± 4.0	
No	17	100		92.3 ± 7.4		86.2 ± 9.1	
D19MRD^†^			0.248		0.688		0.793
<10^-4^	66	93.7 ± 3.0		89.0 ± 3.9		79.9 ± 5.3	
≥10^-4^	70	86.8 ± 4.6		84.2 ± 5.0		79.4 ± 5.3	
D46MRD^†^			0.222		0.135		0.157
<10^-4^	104	92.1 ± 2.7		89.2 ± 3.4		80.8 ± 4.2	
≥10^-4^	17	78.8 ± 11.0		72.2 ± 11.9		66.7 ± 12.2	
Clinical type			0.578		0.88		0.929
Varicella	106	89.6 ± 3.2		86.9 ± 3.6		79.9 ± 4.3	
Herpes zoster	32	92.8 ± 4.9		86.9 ± 6.1		80.5 ± 7.2	

^†^Among patients with data available.

D19MRD, minimal residual disease on 19th day of induction chemotherapy; D46MRD, minimal residual disease on 46th day of induction chemotherapy; EFS, event-free survival; OS, overall survival; RFS, recurrence-free survival; WBC, white blood cells.

**Figure 1:**
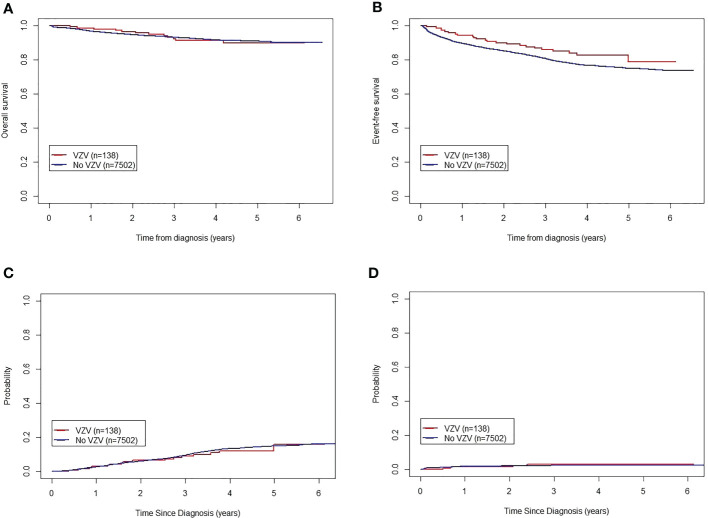
Influence of varicella zoster virus infection on the outcomes of children with acute lymphoblastic leukemia treated according to the CCLG-ALL-2015 protocol. **(A)** Five-year OS of patients with and without VZV infection. **(B)** Five-year EFS of patients with and without VZV infection. **(C)** Five-year CRR of patients with and without VZV infection. **(D)** Five-year death in remission of patients with and without VZV infection. CCCG-ALL-2015, Chinese Children’s Cancer Group-Acute Lymphoblastic Leukemia-2015 trial; CRR, cumulative recurrence rate; EFS, event-free survival; OS, overall survival; VZV, varicella-zoster virus.

All 7,640 patients completed disease evaluation; 3,948 cases entered low-risk treatment, and 3,692 were treated using the intermediate/high-risk protocol. By June 30, 2021, 1,729 patients had only reached continuation therapy 2. The remaining patients had completed the treatment protocol. Twenty-six patients infected with VZV accounted for 18.8% of events, including 16 cases (61.5%) of relapse, 3 patients lost to follow-up (12%), 4 deaths (15%), and 3 (12%) active withdrawals from treatment. In contrast, among the patients without VZV infection, 1,697 patients accounted for 22.2% of events, including 851 (50.1%) cases of relapse, 120 (7.1%) patients lost to follow-up, 169 (10.0%) deaths, 279 (16.4%) active withdrawals from treatment, 50 (2.9%) cases of resistance to treatment, 12 (0.71%) cases of second tumors, 92 (5.4%) passive withdrawals from treatment, and 124 (7.3%) transfers to other medical institutes. Among the patients with and without VZV infection, the 5-year OS rates were 89.8% (95% CI: 84.4–95.6%) and 90.8% (95% CI: 90.0–91.6%), respectively (*P* = 0.80). The 5-year EFS rates of patients with and without VZV infection were 78.8% (95% CI: 69.5–89.3%) and 74.9% (95% CI: 73.7–76.1%), respectively (*P* = 0.09). The 5-year CRRs for patients with and without VZV infection were 15.8% ± 9.2% and 15.0% ± 1.0%, respectively (*P* = 0.71). The 5-year rates of death in remission for patients with and without VZV infection were 3.0% ± 2.9% and 2.3% ± 0.3%, respectively (*P* = 0.65). There were no significant differences between the groups (**Figure 1**).

## Discussion

In this analysis of 7,640 children with ALL enrolled in the CCCG-ALL-2015 trial, the incidence of VZV infection was 1.8% which was significantly higher than that in the general population. Xu et al. (2021) reported that the incidence of varicella in Hangzhou, Zhejiang Province, in 2019 was 120 cases/100,000 population (0.12%). However, compared with other international collaborative pediatric ALL cohorts (Zawitkowska et al., 2020) the treatment according to the CCCG-ALL-2015 protocol did not increase the incidence of VZV infection in patients with ALL.

Age ≥ 10 years was independently associated with VZV infection. This could be explained by the fact that in China, children received one dose of the vaccine when they were younger than 4 years of age and their protection from VZV infection therefore wore off with time (Hu QQ et al., 2022). In this study, only approximately half of the patients received a vaccine at a young age, with the rest not receiving a vaccine at all. Patients older than 10 years either received a VZV vaccine or were not easily infected with VZV. It is well known that one dose of the VZV vaccine infers about 80% protection and 2 doses infer nearly 100% protection (Hu QQ et al., 2022). Our result also supported this conclusion. Based on our results, children are recommended to take 2 doses of the VZV vaccine to gain stronger protection. The *E2A/PBX1* fusion gene was also shown to be independently associated with VZV infection; however, the reason why remains unclear. We suspected that it may be due to the subtype itself. It is well known that different subtypes result in different outcomes in ALL patients. Patients harboring the *E2A/PBX1* fusion gene are classified as being at intermediate risk, and when treated with an intensive treatment they show a comparative outcome to patients with the *ETV6-RUNX1* fusion gene (Pui et al., 2003; Moorman et al., 2010; Stary et al., 2014; Hu et al., 2016). However, people don’t clearly know why and how the subtypes influence the treatment results. Lee et al. (2021) recently identified a germline risk locus located in an intergenic region between *BCL11A* and *PAPOLG* in *E2A/PBX1* ALL patients, indicating that the interplay between germline variants and somatic genomic abnormalities determines the specific ALL subtype. The relationship between *E2A/PBX1* fusion gene subtype and VZV infection requires further exploration in the future.

Only 4 out of 7,640 patients (0.05%) died from VZV infection. Caniza MA et al. (Caniza et al., 2012) retrospectively analyzed the records of 15 pediatric ALL cohorts, studied between 1984 and 2008, that included 4,882 cases from Japan and Taiwan. The authors found that 2 (0.04%) children died of VZV infection (Caniza et al., 2012), a mortality rate similar to that in the present study. However, this rate was lower than the mortality rate reported in North America (3/11,558; 0.026%), whereas the rate in Europe (15/18,688; 0.080%) was higher (Caniza et al., 2012). The reasons for the differences in mortality were not reported in the aforementioned study (Caniza et al., 2012). The human leukocyte antigen-related *HCP5* gene *HCP5* and *IL-10* gene polymorphisms are associated with ethnicity and age in patients with VZV infection (Cho et al., 2007; Crosslin et al., 2015). Caniza et al. (2012) retrospectively analyzed the causes of death in 20 patients who died of VZV infection. Except for 2 cases where the cause of death was unknown, the other 18 patients died of complications secondary to VZV infection, including hepatitis, pneumonia, myocarditis, multiple organ failure, and disseminated intravascular coagulation (Caniza et al., 2012). Their results (Caniza et al., 2012) were similar to ours. In our cohort, 4 patients died of complex VZV infections.

Among 138 patients with VZV infection, the incidence of simple VZV infection was 79.7%, and the incidence of complex VZV infection was 20.3%. The incidence of complex VZV infection was significantly higher (55.6%) in children with exposure history. The 4 children who died of VZV infection had complex VZV infection, indicating that the risk of environmental exposure to VZV is greater for immunocompromised children and isolation should be enforced. Manistarski et al. (2018) suggested extending the isolation time of pediatric immunocompromised ALL patients to reduce the risk of complex infections. Zawitkowska et al. (Zawitkowska et al., 2020) also found higher VZV proneness during the maintenance stage of treatment, presumably because patients were not isolated during this period and, therefore, the corresponding risk of infections increased. Our findings also suggested that VZV infection is more common during the continuing treatment stage, and that being pre-vaccinated or undergoing treatment with IVIG/acyclovir was not correlated with the incidence of complex VZV infection. However, a history of contact with VZV significantly increased the occurrence of complex VZV. Among the 10 patients who died, 7 were infected with VZV during the continuation stage of ALL treatment, 2 were infected with VZV during the completing stage of ALL treatment and 1 was infected with VZV during the consolidation stage of ALL treatment. Regarding cause of death, 4 patients died from complex VZV, 5 from relapse, and 1 from treatment-related. This occurrence may be associated with prolonged immunodeficiency and severe suppression of immune function during chemotherapy. Environmental monitoring and protection of these patients should be enforced to avoid exposure to infectious agents.

In our cohort, VZV infection did not affect OS or ALL recurrence. Children with ALL and VZV infection had similar 5-year OS, EFS, and CRR to those without VZV infection. The Polish National ALL Cooperative Group reported that, although the incidence of VZV infection was higher in children with ALL than in the general population, none of the patients with ALL with VZV infection died (Xu et al., 2021). Their results also indicated that VZV infection does not affect the prognosis of children with ALL (Xu et al., 2021). The Polish study also explained why vaccination before the onset of ALL had no effect on the prevention and control of VZV infection and prognosis, mainly because of weaker humoral immunity corresponding to the immunosuppressed state of the patient due to disease and chemotherapy. Caniza et al. (2012) reported one case of death from vaccination in 35,128 children with ALL. The investigators did not find any significant effect of vaccination on ALL deaths; therefore, on balance, vaccination is not recommended for children with ALL. Furthermore, some studies indicated that VZV vaccination is only 50% effective in patients with ALL (Cakir et al., 2012; Zawitkowska et al., 2020). Thus, immunocompromised children, such as those with ALL who are undergoing treatment or whose immunity has not been restored after treatment, should avoid exposure to VZV, even after being vaccinated (Kim et al., 2014; Kim et al., 2016; Kennedy and Gershon, 2018). However, many researchers advocate for vaccination in immunocompromised patients (Pui et al., 2017) with some studies suggesting that vaccination during maintenance therapy is both safe and effective (Kennedy and Gershon, 2018). A US study (Zawitkowska et al., 2020) showed that, since VZV vaccination has been promoted, the overall incidence of varicella in the US has decreased significantly. This reduced exposure to ALL, and consequently also reduced morbidity in children with ALL. In our cohort, 3818 patients (3818/7640,49.9%) received only one dose of the vaccine. The rate of one dose of varicella attenuated live vaccine (VarV) in children aged 1-14 years in China has reached 50% (Hu QQ et al., 2022). According to the Chinese VarV instructions, children aged 1-12 can receive one dose of basic immunization, as well as one dose of booster immunization when necessary based on the local epidemic prevention and control needs. Therefore, in the past, one dose of immunization was usually used for VarV vaccinations (Hu QQ et al., 2022). According to the report (Stary et al., 2014), the efficacy of one-dose vaccines is about 81% and that of two-dose vaccines is about 92%. However, we lack the appropriate method for detecting successful vaccination. At present, in China, it is generally believed that the protection rate of the two doses of the vaccine is high, but the antibody titer is different due to the differences in the body’s reaction and protection rate. The ALL patients were in immune suppression due to primary disease and chemotherapy treatment. Therefore, universal two-dose vaccination of healthy children is recommended to reduce the incidence of VZV infection and associated complications during ALL treatment. We therefore call for an evaluation of vaccination effectiveness.

In conclusion, we found a significantly higher incidence of VZV infection in children with ALL than in the general population. Age ≥10 years, *E2A/PBX1* gene fusion, and environmental exposure to VZV predisposed patients to developing complex VZV infection, which directly increased their mortality rates. Protective measures, such as avoiding contact with individuals with VZV infection are suggested for immunocompromised children with ALL, even after vaccination.

## Data availability statement

The datasets presented in this study can be found in online repositories. The names of the repository/repositories and accession number(s) can be found in the article/**Supplementary Material**.

## Ethics statement

This study was approved by the institutional review board of each participating center. Written informed consent to participate in this study was provided by the participants’ legal guardian/next of kin.

## Author contributions

SH, SS, and JT conceptualized and designed the study. WG and SH performed the literature review. PFX, JG, XMG, AWL, YYH, YZ, JHC, XWZ, BQQ, AGL, LCY, JSZ, ZL, XT, YX, LH, XDW, FZ, and LZW acquired the data. WG and JC performed the statistical analyses. WG and SH drafted the manuscript. JC created the figures. All authors were involved in data interpretation, and provided critical revision of the manuscript for important intellectual content.

## Funding

This work was supported by the grants from National Science Foundation of China (81970163 and 82170218); Jiangsu Key project (BE2021654); Suzhou Key Project (GSWS2020039); and National Clinical Research Center for Hematological Disorders (2020ZKPB02).

## Acknowledgments

We are grateful for the support from VIVA-China Children’s Cancer Foundation. The manuscript was edited for language by Editage.

## Conflict of interest

The authors declare that the research was conducted in the absence of any commercial or financial relationships that could be construed as a potential conflict of interest.

## Publisher’s note

All claims expressed in this article are solely those of the authors and do not necessarily represent those of their affiliated organizations, or those of the publisher, the editors and the reviewers. Any product that may be evaluated in this article, or claim that may be made by its manufacturer, is not guaranteed or endorsed by the publisher.
