# Supplementary material for: A prospective multicenter study on varicella-zoster virus infection in children with acute lymphoblastic leukemia
Source: Front Cell Infect Microbiol. 2022 Nov 11;12:981220. doi: 10.3389/fcimb.2022.981220 (PMC9691833; doi:10.3389/fcimb.2022.981220)
Supplement: Supplementary file 1 [file Table_1.docx]

Supplementary Material

# Supplementary Figures and Tables

## Supplementary Tables

| Supplemental Table 1 The multivariate analysis of factors related to VZV | | | | | |
| --- | --- | --- | --- | --- | --- |
| Factor | OR | SE | Wald | β | P value |
| Age | 0.537 | 0.229 | 7.380 | -0.621 | 0.007 |
| Risk | 1.068 | 0.185 | 0.127 | 0.066 | 0.722 |
| *E2A/PBX1* | 2.474 | 0.284 | 10.138 | 0.906 | 0.001 |

VZV, varicella zoster virus.

|  |  |  | | Supplemental Table 2 Analysis of factors related to relapse | | | | | | |
| --- | --- | --- | --- | --- | --- | --- | --- | --- | --- | --- |
| Factor | | |  | | Relapse (N=16, %) | No relapse (N=122, %) | OR | 95% CI for OR | | P |
|  |  |  |  |  |  |  |  | Lower | Upper |  |
| Age(year) | | |  | |  |  | 0.596 | 0.190 | 1.868 | 0.564 |
|  | | | 1-10 | | 11 (68.75) | 96(78.69) |  |  |  |  |
|  | | | ＞10 | | 5(31.25) | 26(21.31) |  |  |  |  |
| Gender | | |  | |  |  | 2.304 | 0.703 | 7.551 | 0.159 |
|  | | | Male | | 12(75.00) | 69(56.56) |  |  |  |  |
|  | | | Female | | 4(25.00) | 53(43.44) |  |  |  |  |
| Immunotype | | |  | |  |  | 0.297 | 0.082 | 1.080 | 0.133 |
|  | | | B-ALL | | 12(75.00) | 111(91.74) |  |  |  |  |
|  | | | T-ALL | | 4(25.00) | 11(8.26) |  |  |  |  |
| WBC(×10^9/L) | | |  | |  |  | 0.539 | 0.171 | 1.697 | 0.458 |
|  | | | <50 | | 11 (68.75) | 98(80.33) |  |  |  |  |
|  | | | ≥50 | | 5(31.25) | 24(19.67) |  |  |  |  |
| Initial risk | | |  | |  |  | 0.399 | 0.131 | 1.216 | 0.097 |
|  | | | Low | | 5(31.25) | 65(53.28) |  |  |  |  |
|  | | | Intermediate/High | | 11 (68.75) | 57(46.72) |  |  |  |  |
| Ultimate risk | | |  | |  |  | 0.356 | 0.109 | 1.165 | 0.078 |
|  | | | Low | | 4(25.00) | 59(48.36) |  |  |  |  |
|  | | | Intermediate/High | | 12(75.00) | 63(51.64) |  |  |  |  |
| Karyotype | | |  | |  |  | 0.687 | 0.145 | 3.251 | 0.905 |
|  | | | >50 | | 2(12.5) | 21(17.21) |  |  |  |  |
|  | | | Others | | 14(87.5) | 101(82.79) |  |  |  |  |
| *TEL/AML1* | | |  | |  |  | 0.412 | 0.051 | 3.323 | 0.643 |
|  | | | Positive | | 1(6.25) | 17(13.93) |  |  |  |  |
|  | | | Negative | | 15(93.75) | 105(86.07) |  |  |  |  |
| *E2A/PBX1* | | |  | |  |  | 0.476 | 0.059 | 3.865 | 0.768 |
|  | | | Positive | | 1(6.25) | 15(12.30) |  |  |  |  |
|  | | | Negative | | 15(93.75) | 107(87.70) |  |  |  |  |
| *BCR/ABL1* | | |  | |  |  | 1.560 | 0.171 | 14.267 | 0.530 |
|  | | | Positive | | 1(6.25) | 5(4.10) |  |  |  |  |
|  | | | Negative | | 15(93.75) | 117(95.90) |  |  |  |  |
| *MLL-r* | | |  | |  |  | 1.134 | 1.066 | 1.207 | 1.000 |
|  | | | Positive | | 0(0.00) | 3(2.46) |  |  |  |  |
|  | | | Negative | | 16(100.00) | 119(97.54) |  |  |  |  |
| Treatment delay | | |  | |  |  | 0.835 | 0.170 | 4.091 | 1.000 |
|  | | | Yes | | 14(87.5) | 109(89.34) |  |  |  |  |
|  | | | No | | 2(12.5) | 13(10.66) |  |  |  |  |
| D19MRD* | | |  | |  |  | 0.804 | 0.281 | 2.299 | 0.684 |
|  | | | <10^-4 | | 7(43.75) | 59(49.17) |  |  |  |  |
|  | | | ≥10^-4 | | 9(56.25) | 61(50.83) |  |  |  |  |
| D46MRD* | | |  | |  |  | 0.346 | 0.095 | 1.264 | 0.210 |
|  | | | <10^-4 | | 10(71.43) | 94(87.85) |  |  |  |  |
|  | | | ≥10^-4 | | 4(28.57) | 13(12.15) |  |  |  |  |
| Severity | | |  | |  |  | 1.117 | 0.295 | 4.223 | 1.000 |
|  | | | Skin only | | 13(81.25) | 97(79.51) |  |  |  |  |
|  | | | Complicated | | 3(18.75) | 25(20.49) |  |  |  |  |
| Clinical type | | |  | |  |  | 0.894 | 0.267 | 2.990 | 1.000 |
|  | | | Varicella | | 12(75.00) | 94(77.05) |  |  |  |  |
|  | | | Zoster | | 4(25.00) | 28(22.95) |  |  |  |  |

^*^data available

WBC, white blood cells; D19MRD, minimal residual disease on 19th day of induction chemotherapy; D46MRD, minimal residual disease on 46th day of induction chemotherapy.

| Supplemental Table 3 The multivariate analysis of factors related to EFS | | | | | |
| --- | --- | --- | --- | --- | --- |
| Factor | β | SE | Wald | OR | P value |
| Immunotype | 1.068 | 0.488 | 4.784 | 2.909 | 0.029 |
| WBC | 0.561 | 0.465 | 1.454 | 1.753 | 0.228 |
| BCR/ABL1 | -1.219 | 0.644 | 3.585 | 0.296 | 0.058 |

WBC, white blood cells.
